# Supplementary material for: Rapid Tryptophan Assay as a Screening Procedure for Quality Protein Maize
Source: Molecules. 2024 Sep 12;29(18):4341. doi: 10.3390/molecules29184341 (PMC11434058; doi:10.3390/molecules29184341)
Supplement: Supplementary file 1 [file molecules-29-04341-s001.zip › molecules-3116052-supplementary.pdf]

molecules

MDPI

Article

Rapid tryptophan assay as a screening procedure for quality protein maize

Gabi Drochioiu [1,*], Elena Mihalcea [1], Jeanclaude Lagobo [1,2] and Catalina-Ionica Ciobanu [3]

[1] Faculty of Chemistry, Alexandru Ioan Cuza University of Iasi, 11 Carol I, 700506 Iasi, Romania; lenuta_mihalcea@yahoo.com (E.M.); lagobojeanclaude@gmail.com (J.L.)
[2] Environmental Science Laboratory (LSE), Nangui-Abrogoua University, Abidjan 02 BP 801, Côte d'Ivoire
[3] Integrated Centre of Environmental Science Studies in the North East Region—CERNESIM Centre, Institute of Interdisciplinary Research, Alexandru Ioan Cuza University of Iasi, 11 Carol I, 700506 Iasi, Romania; catalina.ciobanu@uaic.ro
* Correspondence: gabidr@uaic.ro

Table S1. Tryptophan levels of representative maize samples determined both in enzyme hydrolyzed solutions [27] and in the biuret supernatants.

Sample	16-hour enzymatic hydrolysis [1]	Proposed micro-method [1]	Mean tryptophan level [1]	Difference between tryptophan levels
HT-17/81	0.37	0.39	0.380	0.02
HT-38/80	0.37	0.35	0.360	-0.02
HD 22/81	0.43	0.45	0.440	0.02
L-2	0.43	0.46	0.445	0.03
HT-39/88	0.45	0.42	0.435	-0.03
HD-18/87	0.47	0.45	0.460	-0.02
L-3	0.47	0.46	0.465	-0.01
HT-40/89	0.48	0.44	0.460	-0.04
L-1	0.49	0.46	0.475	-0.03
L-5	0.49	0.47	0.480	-0.02
HT-34/89	0.5	0.5	0.500	0
L-4	0.5	0.47	0.485	-0.03
O-65/88	0.69	0.71	0.700	0.02
O-26/86	0.72	0.77	0.745	0.05
O-53/87	0.73	0.79	0.760	0.06
O-37/88	0.74	0.77	0.755	0.03
O-67/86	0.74	0.8	0.770	0.06
O-42/88	0.75	0.78	0.765	0.03
O-21/86	0.77	0.79	0.780	0.02
O-43/88	0.77	0.8	0.785	0.03
O-21/86	0.77	0.81	0.790	0.04
O-13/87	0.81	0.83	0.820	0.02
Lizin 125	0.81	0.8	0.805	-0.01
O-415	0.82	0.83	0.825	0.01
O-18/87	0.82	0.86	0.840	0.04

[1] % tryptophan in crude protein.

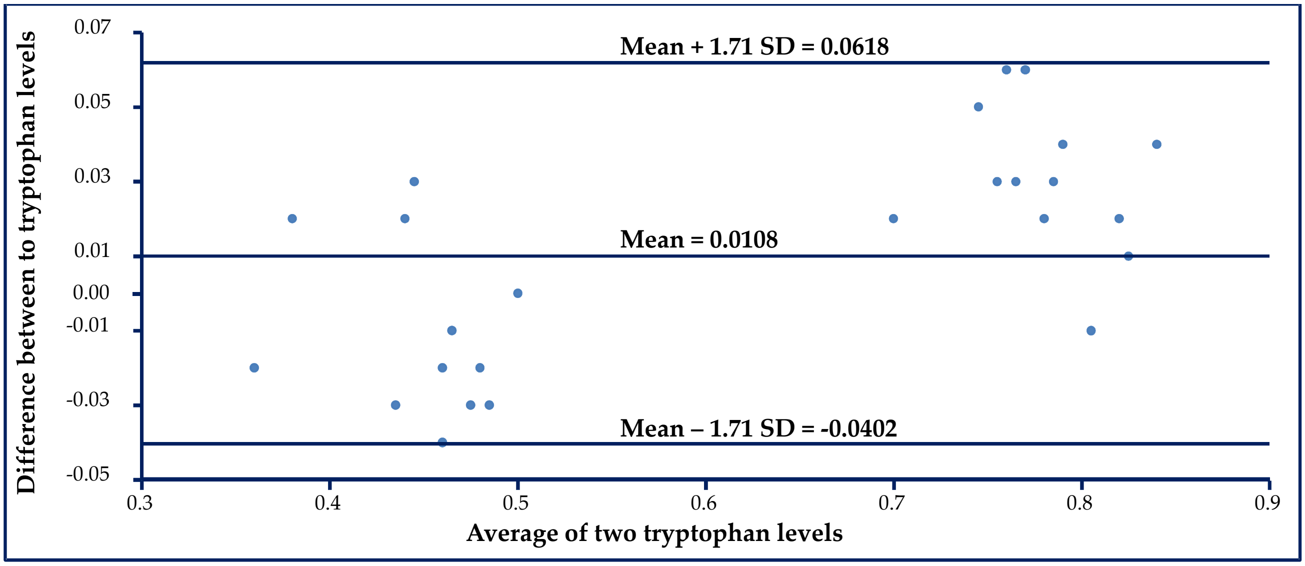

Figure S1. Agreement between two tryptophan measurements (Bland-Altman plot).

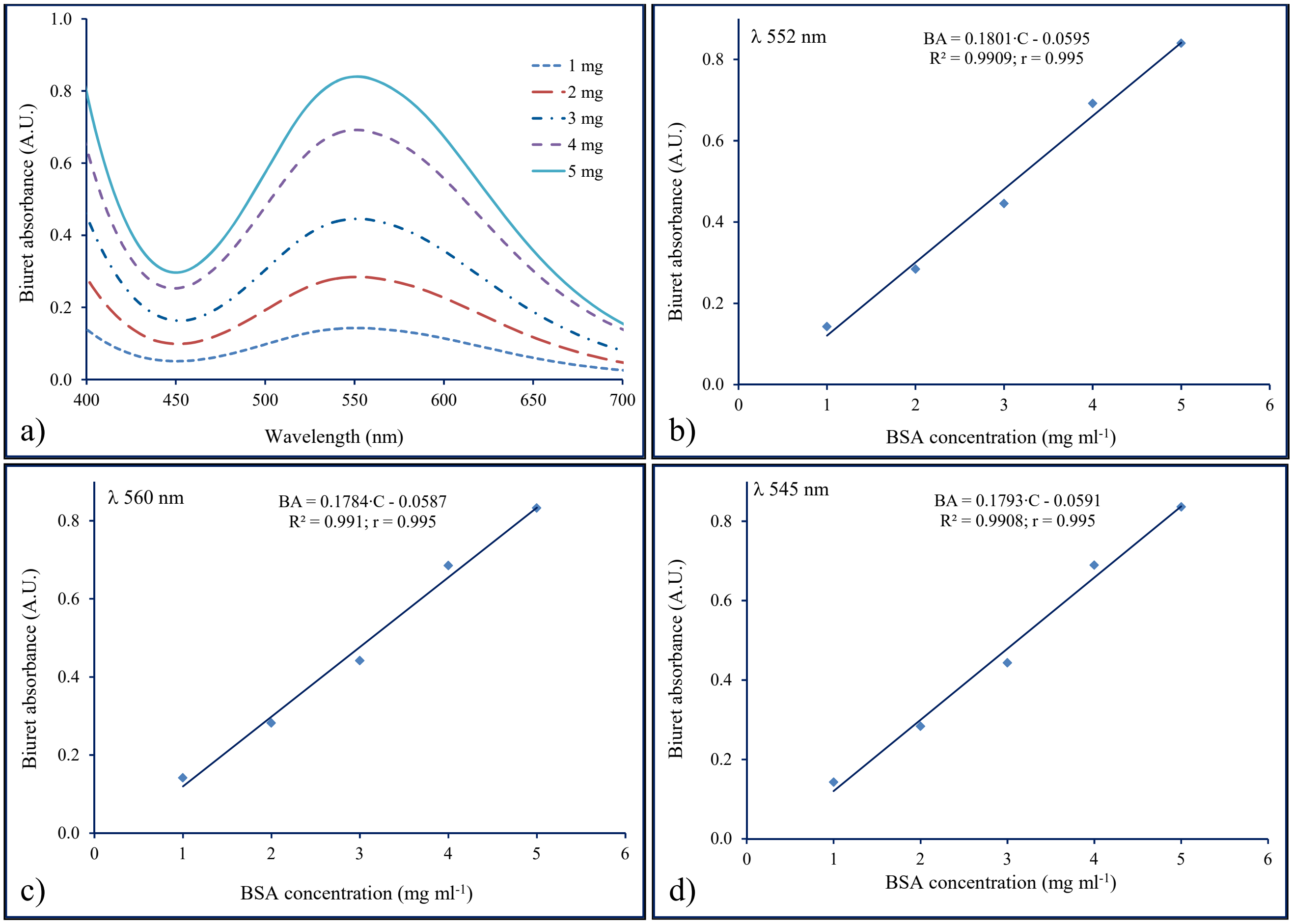

Figure S2. Standard curves in protein determination using bovine serum albumin (BSA). Here, a) absorbance spectra of different concentrations of BSA (1-5 mg mL^{-1}); b) calibration curve with absorbance values at 552 nm; c) calibration curve with absorbance values at 560 nm; d) calibration curve with absorbance values at 545 nm, respectively.

Table S2. Chemical composition of 25 representative maize samples illustrating their nutritional quality.

Sample *	Tryptophan (mg % in DM)	Crude protein (% in DM)	Zein (% in DM)	Lysine (mg% in DM)	Lysine (% in crude protein)	Tryptophan (% in crude protein)
HD 22/81	35.09	8.16	2.22	179.5	2.20	0.43
HT-39/88	39.60	8.80	2.34	205.9	2.34	0.45
HT-40/89	44.54	9.28	2.54	225.5	2.43	0.48
HD-18/87	44.98	9.57	2.83	231.6	2.42	0.47
L-2	45.79	10.65	3.61	252.4	2.37	0.43
HT-38/80	47.43	12.82	5.89	251.3	1.96	0.37
L-5	50.52	10.31	3.39	207.2	2.01	0.49
L-3	50.62	10.77	3.39	228.3	2.12	0.47
HT-34/89	51.20	10.24	3.40	245.8	2.40	0.50
L-1	51.35	10.48	3.28	236.8	2.26	0.49
L-4	57.20	11.44	4.17	268.8	2.35	0.50
HT-17/81	59.09	15.97	6.68	300.2	1.88	0.37
O-21/86	59.44	7.72	1.10	271.7	3.52	0.77
O-65/88	65.69	9.52	1.90	306.5	3.22	0.69
O-18/87	70.11	8.55	1.24	312.9	3.66	0.82
O-67/86	73.48	9.93	1.83	329.7	3.32	0.74
O-415	75.44	9.20	1.14	346.8	3.77	0.82
O-37/88	77.33	10.45	2.19	344.9	3.30	0.74
O-43/88	85.09	11.05	1.35	375.7	3.40	0.77
O-53/87	85.41	11.70	1.75	390.8	3.34	0.73
Lizin 125	87.97	10.86	2.28	366.0	3.37	0.81
O-42/88	88.05	11.74	1.24	403.9	3.44	0.75
O-21/86	97.17	12.62	1.45	421.5	3.34	0.77
O-13/87	99.14	12.24	1.35	432.1	3.53	0.81
O-26/86	100.36	13.94	2.42	446.1	3.20	0.72

* O – *opaque*-2; Lizin - *opaque*-2; L – inbred line; HD – double hybrid; HT – trilinear hybrid.

From the relationship between lysine and tryptophan in the maize seeds (Figure S4), it is believed that a tryptophan value could serve as a single parameter for maize evaluation. This study proves the feasibility of rapid tryptophan analysis and the importance of the correlation between tryptophan and lysine.

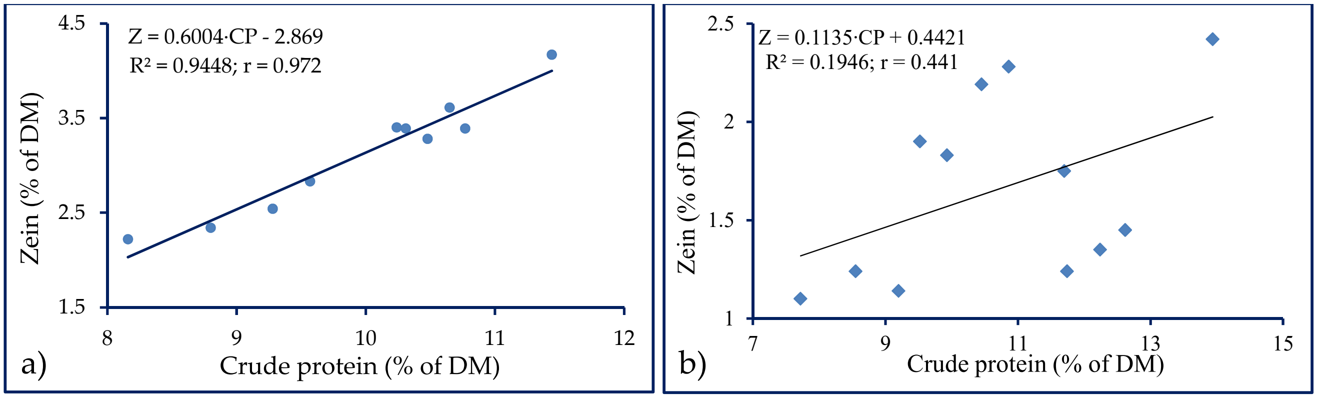

Figure S3. The relationship between zein and crude protein in some real samples. Here, a) the strong relationship between zein and crude protein in normal maize; b) the QPM samples contain low amounts of zein.

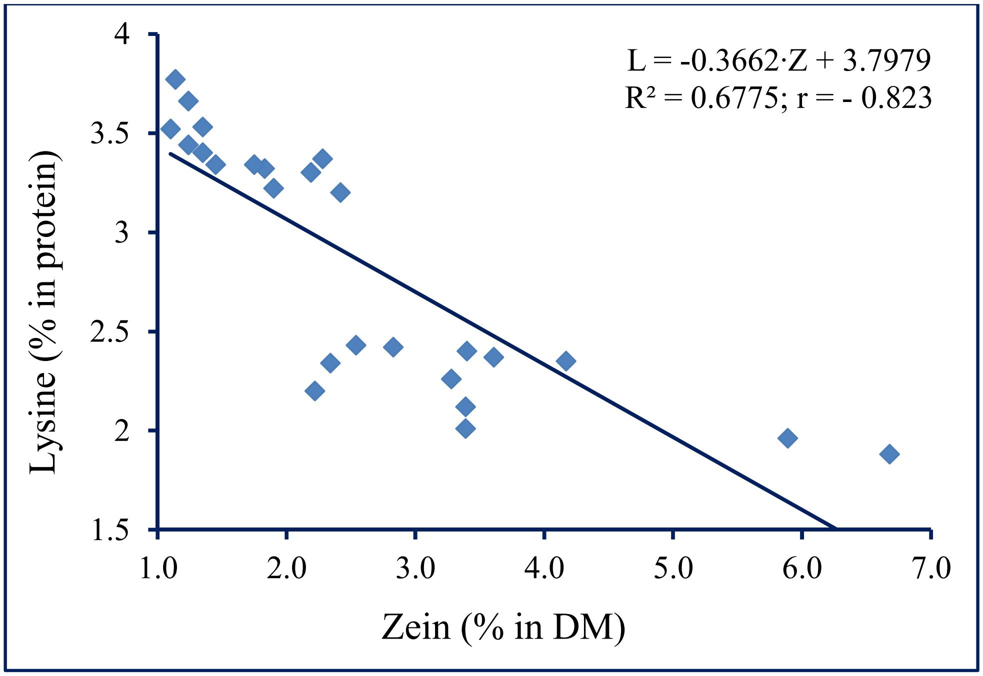

Figure S4. Relationship between lysine and zein in maize samples studied.

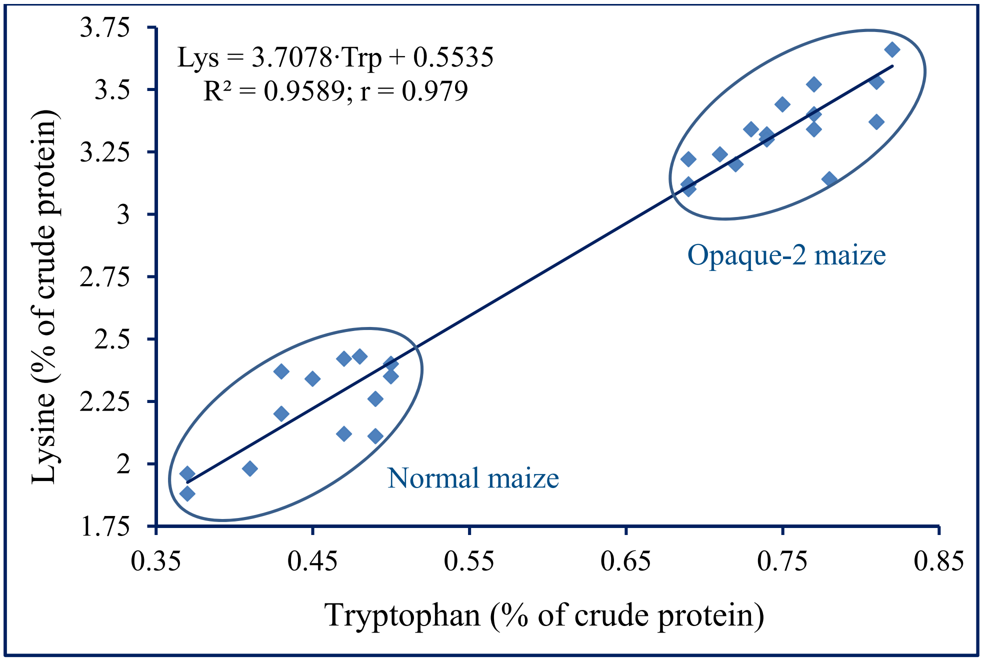

Figure S5. Relationship between lysine and tryptophan content of 30 different maize samples.
